# Improved diagnostic accuracy of readout-segmented echo-planar imaging for peripheral zone clinically significant prostate cancer: a retrospective 3T MRI study

**DOI:** 10.1038/s41598-024-53898-0

**Published:** 2024-02-08

**Authors:** M. Deforche, Y. Lefebvre, R. Diamand, M. A. Bali, M. Lemort, N. Coquelet

**Affiliations:** 1 Radiology Department, Institut Jules Bordet, HUB–University Hospital of Brussels, 90 Rue Meylemeersch, 1070 Brussels, Belgium; 2 Urology Department, Institut Jules Bordet, HUB–University Hospital of Brussels, Brussels, Belgium

**Keywords:** Cancer, Prostate

## Abstract

This study compares the readout-segmented echo-planar imaging (rsEPI) from the conventional single-shot EPI (ssEPI) diffusion-weighted imaging (DWI) for the discrimination of patients with clinically significant prostate cancer (csPCa) within the peripheral zone (PZ) using apparent diffusion coefficient (ADC) maps and pathology report from magnetic resonance imaging (MRI)-targeted biopsy. We queried a retrospective monocentric database of patients with targeted biopsy. csPCa patients were defined as an International Society of Urological Pathology grade group ≥ 2. Group-level analyses and diagnostic accuracy of mean ADC values (ADC_mean_) within the tumor volume were assessed from Kruskal–Wallis tests and receiving operating characteristic curves, respectively. Areas under the curve (AUC) and optimal cut-off values were calculated. 159 patients (105 rsEPI, 54 ssEPI; mean age ± standard deviation: 65 ± 8 years) with 3T DWI, PZ lesions and targeted biopsy were selected. Both DWI sequences showed significantly lower ADC_mean_ values for patients with csPCa. The rsEPI sequence better discriminates patients with csPCa (AUC_rsEPI_ = 0.84, AUC_ssEPI_ = 0.68, *p* < 0.05) with an optimal cut-off value of 1232 μm^2^/s associated with a sensitivity–specificity of 97%-63%. Our study showed that the rsEPI DWI sequence enhances the discrimination of patients with csPCa.

## Introduction

Multiparametric magnetic resonance imaging (mpMRI) for the detection of prostate cancer (PCa) has become fundamental to better select patients with clinically significant prostate cancer (csPCa) and guide them towards MRI-targeted biopsy (for a review, see, e.g.,^1^).

Prostate imaging-reporting and data system (PI-RADS) guidelines^2^ state that the determinant MR acquisition sequence for the detection of PCa in the peripheral zone (PZ) –which represents about 65% of all PCa^3^—is the diffusion-weighted imaging (DWI) and its derived apparent diffusion coefficient (ADC) map. Although the PI-RADS guidelines indicate the scoring procedure of a lesion based on the visual assessment of its signal intensity, these guidelines, to date, do not yet include quantitative features based on the ADC maps.

Over the last year, several studies have shown that ADC values of PZ-located lesions (measured from several metrics, such as mean/median ADC or ADC ratio) inversely correlated with the International Society of Urological Pathology (ISUP) grade group (and thus the aggressiveness of tumors)^4–6^ (for reviews, see, e.g.,^7,8^). In addition, efforts have been made to compute optimal ADC thresholds that best differentiate patients according to their clinical status, in particular patients with csPCa from others (see, e.g.,^9^).

Critically, the DWI sequence used in the vast majority of these studies is the single-shot echo-planar imaging (ssEPI), which fills the *k*-space in a single shot. More recently, another DWI sequence, the readout-segmented echo-planar imaging (rsEPI), has gained more attention and proposed to segment the *k*-space into non-overlapping segments in the readout direction. This approach has shown to be less prone to magnetic-susceptibility artifacts and T2* blurring compared to the conventional ssEPI^10^. Although some studies already demonstrated an improved image quality of the rsEPI DWI compared to the standard ssEPI for PCa imaging^11,12^, its impact on ADC values remains to be settled. In particular, no study has investigated to what extent the rsEPI better discriminates patients with csPCa than the classical ssEPI based on ADC values.

Thus, the aim of this study is to compare the diagnostic accuracy of the rsEPI compared to the classical ssEPI in the differentiation of patients with csPCa by correlating ADC maps measurements and pathology results of documented MRI-targeted biopsy.

## Materials and methods

### Study population

This retrospective study was approved by the Institut Jules Bordet ethics committee (CE3462). A written informed consent was waived by the institutional review board of the Institut Jules Bordet. All methods were performed in accordance with the relevant guidelines and regulations.

We queried a retrospective database (March 2016–December 2021) of 544 patients with targeted prostate biopsies performed within our institution. This database includes biopsy-naïve patients only for whom mpMRI was indicated for a clinical suspicion of PCa based on the prostate-specific antigen (PSA) level (> 3 ng/ml) and/or the PSA density (> 0.15 ng/ml/cc, with prostate volume computed by ultrasound during the clinical examination performed by the urologist) and/or a suspicious digital rectal exam (DRE). Our exclusion criteria were: (i) MRI performed either on a 1.5T system or outside the institution, (ii) MRI performed with an additional endorectal coil, (iii) lesions with PI-RADS score 1 or 2 (as, following the EAU guidelines^13^, no biopsy is performed for patients with PI-RADS score 1 or 2, thus no standard of reference was available), (iv) lesions not located within the PZ (as DWI and ADC are the dominant sequences only for PZ lesions^2^), (v) insufficient ADC map quality due to artifacts after careful revision by trainee and experienced radiologists (no PI-QUAL score retrospectively assigned to support insufficient image quality), (vi) no biopsy (or only one biopsy) in the target lesion, (vii) history of pathologically proven PCa, and (viii) insufficient details in the pathology reports.

### Imaging acquisition technique

Images were acquired on a 3.0 Tesla (3T) MRI scanner (MAGNETOM Skyra Fit; Siemens Healthineers, Erlangen, Germany) with the patients in supine position using a phased array surface coil.

According to PI-RADS guidelines, the mpMRI acquisition protocol consisted of (i) a turbo spin-echo pulse T2-weighted (T2w) high-resolution sequence in the three planes, (ii) an axial DWI sequence (see paragraph below for detailed characteristics of the two DWI sequences) and the associated ADC map computed from monoexponential fitting using the lowest and the highest acquired *b*-values, and (iii) an axial dynamic contrast-enhanced imaging before and after intravenous administration of gadoteric acid (Dotarem, Guerbet, Villepinte, France).

Two DWI sequences were compared for the purpose of this study. The first DWI sequence consisted of a rsEPI (RESOLVE; Siemens Healthineers, Erlangen, Germany) and the second DWI sequence is a classical ssEPI. The rsEPI sequence was used for non-corpulent patients (i.e., whenever patient’s weight was below 80 kg) and the ssEPI for corpulent patients (i.e., patient’s weight above 80 kg). Table 1 displays the main MRI parameters of these two DWI sequences.

**Table 1:** MRI parameters of the two DWI sequences.

	rsEPI	ssEPI
TR (ms)	4760	4100
TE (ms)	67	65
FOV (readout ⨉ phase) (mm)	200 ⨉ 200	200 ⨉ 200
Matrix dimension (readout ⨉ phase)	150 ⨉ 150	152 ⨉ 152
Resolution (mm^2^)	1.33	1.32
Slice thickness (mm)—interslice gap (%)	3 mm–10%	3 mm–10%
Number of slices	20	20
*b*-values (averages) (s/mm^2^)	0(1)—1200(5)	50(2)—400(3)—800(100)
Calculated *b*-value (s/mm^2^)	1600	1400
PI—acceleration factor	GRAPPA—R = 2	GRAPPA—R = 2
Partial Fourier (readout—phase)	6/8—n.a	n.a.—6/8
Number of readout segments	5	n.a
Fat saturation	SPAIR	SPAIR
EPI factor	98	152
Echo spacing (ms)	0.38	1.06
Bandwidth (Hz/px)	877	1028
Acquisition time (min:sec)	6:22	4:28

rsEPI, readout-segmented echo-planar imaging; ssEPI, single-shot echo-planar imaging; TR, repetition time; TE, echo time; FOV, field-of-view; EPI, echo-planar imaging; PI, parallel imaging; GRAPPA, generalized autocalibrating partial parallel acquisition; n.a., not applicable; SPAIR, spectral attenuated inversion recovery.

To note, mpMRI images were used to define PI-RADS score (either based on the version 2.0 or version 2.1 according to the time of examination) of the index lesion, while only T2w and DWI images were considered for image analyses (see below).

### Image analysis

All MRI scans were initially analyzed by two dedicated genito-urinary radiologists with respectively eight (Y.L.) and twenty (M.L.) years of experience in prostate MRI interpretation, both fulfilling the criteria of ‘expert’ radiologist according to the ESUI guidelines^14^. For the present study, all MRI scans were retrospectively reviewed by a radiologist fellow (M.D.) under the supervision of the two senior radiologists and the PI-RADS score of the index lesion collected from the written report of the experienced radiologists.

An in-house plug-in (TumourMetrics^15^) based on ImageJ software^16^ used ADC maps to provide a semi-automatic segmentation of the index lesion. Once the tumor identified, it was framed inside a user-defined box in orthogonal views and was automatically segmented based on threshold values. The segmentation was then revised manually using ADC map and T2w images as a guide in order to include the whole tumor volume and to exclude adjacent non-tumor structures. The software determined the whole tumor volume (expressed in mm^3^) and provided a histogram of ADC values of voxels within the whole tumor volume, from which the mean ADC value was extracted (henceforth referred to as “ADC_mean_”).

**Figure 1:**
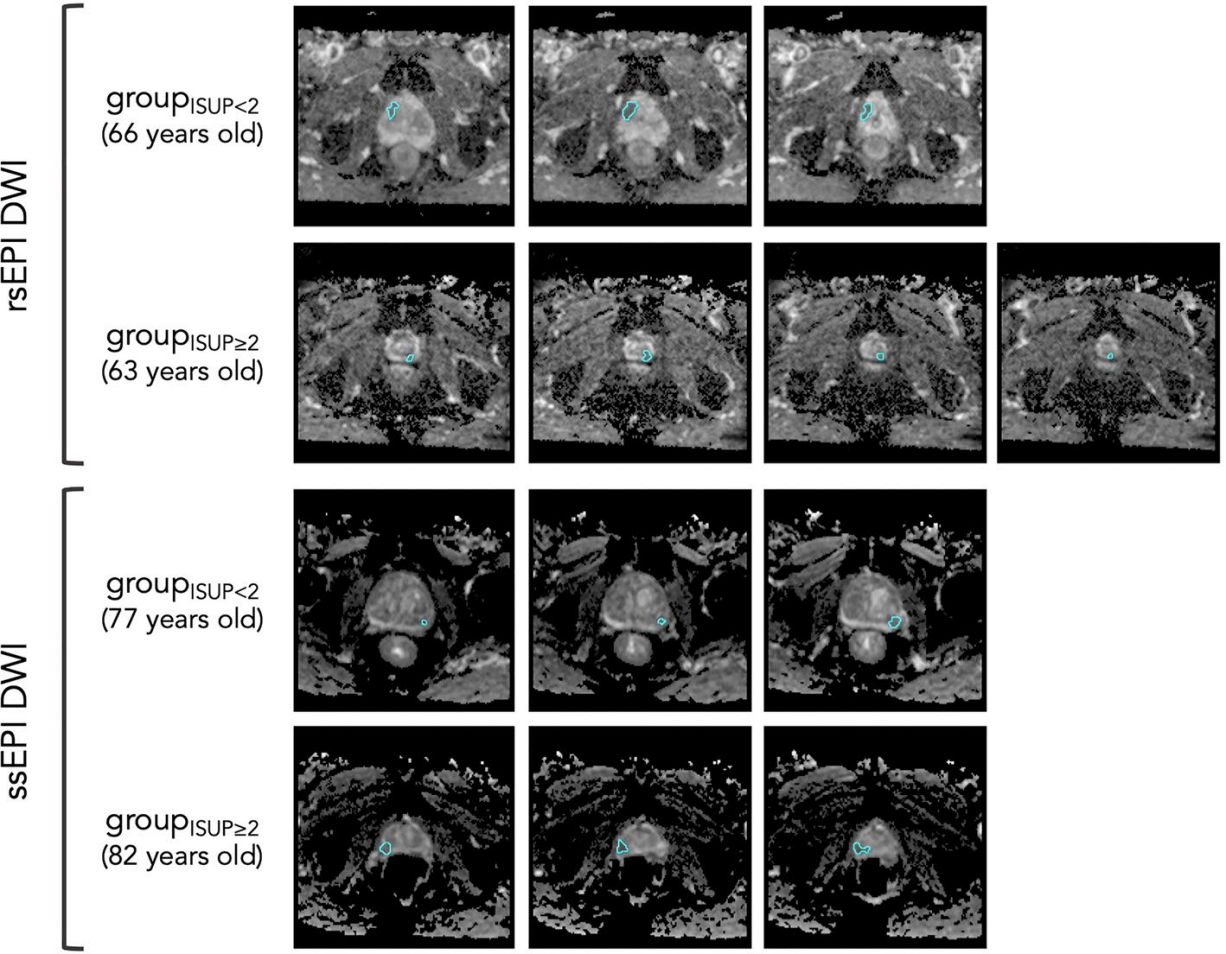
Illustrative example of the two DWI sequences at stake in our study. The two first rows correspond to rsEPI DWI and the two last rows to ssEPI DWI. In each case, the top and bottom rows represent a patient from the group_ISUP<2_ (i.e., a patient having a benign lesion or a ISUP grade group of 1) and a patient from the group_ISUP≥2_ (i.e., a patient with a ISUP grade group ≥ 2), respectively. *Note*: rsEPI = readout-segmented echo-planar imaging, DWI = diffusion-weighted imaging, ssEPI = single-shot echo-planar imaging.

Figure 1 illustrates the two DWI sequences of our study along with the delineation of the index lesion.

### Clinical data

As all index lesions had a PI-RADS score ≥ 3, all patients underwent a targeted biopsy of the index lesion combined with systematic biopsies. Suspicious lesions and prostate contour were manually contoured on T2w or high *b*-value DWI sequence by radiologists and submitted to the biopsy platform. Prostate biopsies were performed transrectally and carried out with the KOELIS system (KOELIS, La Tronche, France) allowing MRI-3D ultrasound images fusion by two urologists dedicated to fusion biopsy (more than 350 MRI-targeted biopsy per year) using Trinity software platforms. A minimum of 3 targeted cores per target were taken combined with concomitant bilateral systematic biopsy; which included at least 6 random cores depending on patient characteristics and physician preferences.

Biopsy pathology results were reported according to the ISUP grade groups. The ISUP grade group of targeted index lesions allows separating our patients into two groups: a first group of patients with csPCA (ISUP grade group ≥ 2, henceforth referred to as “group_ISUP≥2_”) and a second group composed of all other patients (i.e., non-csPCa patients (ISUP grade group of 1) and benign lesions). This group is referred to as “group_ISUP<2_”. Pathological anatomy results of biopsies represent the standard of reference.

### Patients grouping

Our study deals thus with 4 different groups (two ISUP-based groups and two DWI sequences): rsEPI group_ISUP<2_, rsEPI group_ISUP≥2_, ssEPI group_ISUP<2_ and ssEPI group_ISUP≥2_. In order to take into account potential bias selection due to unequal sample size between groups, additional analysis will be performed based on sub-groups of patients matched for age and PSA density.

For all patients, we reported (i) the age (at the time of the examination, in years), (ii) the PSA level (in ng/ml), (iii) the prostate volume based on the MRI (in cc), (iv) the PSA density (in ng/ml/cc), (v) the DRE (soft or indurated), (vi) the diameter of the prostate index lesion (in mm), and (vii) the PI-RADS score of the index lesion. Continuous variables were expressed as median and interquartile range (IQR).

### Statistical analyses

Statistical differences between the two ISUP-based groups for each DWI sequence were assessed with non-parametric Kruskal–Wallis tests. A Bonferroni correction factor of 2 was considered to take into account multiple comparisons.

Diagnostic performance to differentiate group_ISUP<2_ from group_ISUP≥2_ was assessed using receiver operating characteristic (ROC). Area under the curve (AUC) and the standard error of AUC were computed according to the method of DeLong et al.^17^. AUC values were reported with their 95% confidence interval. Optimal cut-off values were calculated using the Youden’ J statistic^18^, and associated with a pair of sensitivity–specificity. Diagnostic accuracies were evaluated for the two DWI sequences. Statistical comparisons between the two AUCs were performed using non-parametric DeLong tests^17^.

All statistical tests were two-tailed and *p*-values below 0.05 were deemed significant.

Statistical analyses were performed using MatlabR2022.

## Results

### Study population characteristics

**Figure 2:**
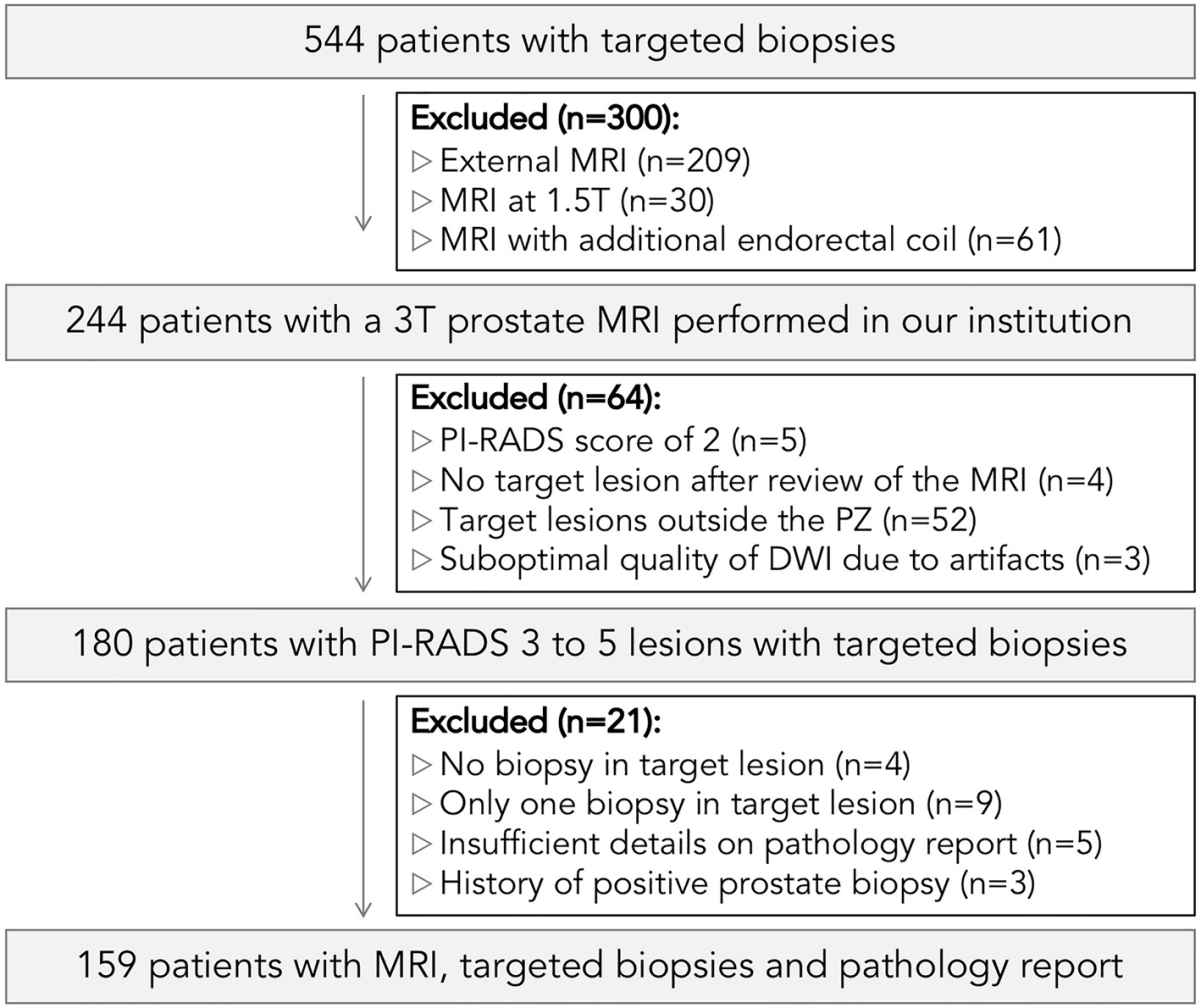
Participant enrollment in the study and exclusion. From 544 patients, 385 were excluded, leading to a final dataset of 159 patients with 3T MRI using a phased array surface coil, targeted biopsies of index lesion located within PZ and pathology report performed within our institution. *Note*: MRI = magnetic resonance imaging, PI-RADS = prostate imaging-reporting and data system, PZ = peripheral zone, DWI = diffusion-weighted imaging.

Out of 544 patients, we selected and subsequently analyzed 159 patients with (i) 3T MRI and targeted biopsy performed within our institution, (ii) targeted lesion located within the PZ, (iii) sufficient ADC map quality, and (iv) sufficient details in the biopsy report. A total of 105 patients underwent the rsEPI DWI sequence (70 for the group_ISUP<2_ and 35 for the group_ISUP≥2_) and 54 patients underwent the ssEPI DWI sequence (32 for the group_ISUP<2_ and 22 for the group_ISUP≥2_). Table 2 reports the demographic data according to the ISUP-based group and the DWI sequence. Figure 2 depicts the flowchart of the study.

**Table 2:** Demographic characteristics of study population according to the DWI sequence and the ISUP grade group. Age, PSA level, PSA density, index lesion diameter and prostate volume on MRI are expressed as median and interquartile range.

	rsEPI (*N* = 105)		ssEPI (*N* = 54)	
	group_ISUP<2_	group_ISUP≥2_	group_ISUP<2_	group_ISUP≥2_
Number	70	35	32	22
Age (years)	63.5 (58–70)	70 (62.5–74.75)	66.5 (58–70.5)	71 (67–73)
PSA (ng/ml)	6 (3.8–8)	8.8 (5.3–11.1)	6 (3.9–9.9)	7.1 (5.3–11.3)
Prostate volume on MRI (cc)	45 (35–67)	38.2 (31.9–51.5)	42 (31–74.4)	36 (29.6–53)
PSA density (ng/ml/cc)	0.12 (0.09–0.17)	0.19 (0.12–0.3)	0.12 (0.11–0.2)	0.21 (0.1–0.34)
DRE (soft/indurated)	58/12	23/12	26/6	8/14
Index lesion diameter (mm)	11.5 (9–13)	15 (10–18)	10.5 (7.5–14)	16 (12–18)
# PI-RADS score 3 (%) # PI-RADS score 4 (%) # PI-RADS score 5 (%)	18 (25.7%) 45 (64.3%) 7 (10%)	1 (2.8%) 15 (42.8%) 19 (54.4%)	5 (15.6%) 21 (65.6%) 6 (18.8%)	0 (0%) 8 (36.4%) 14 (63.6%)

rsEPI, readout-segmented echo-planar imaging; ssEPI, single-shot echo-planar imaging; ISUP, international society of urological pathology; PSA, prostate-specific antigen; MRI, magnetic resonance imaging; DRE, digital rectal examination; PI-RADS, prostate imaging-reporting and data system.

### *ISUP-based between-group comparison of ADC*_*mean*_* for each DWI sequence*

**Figure 3:**
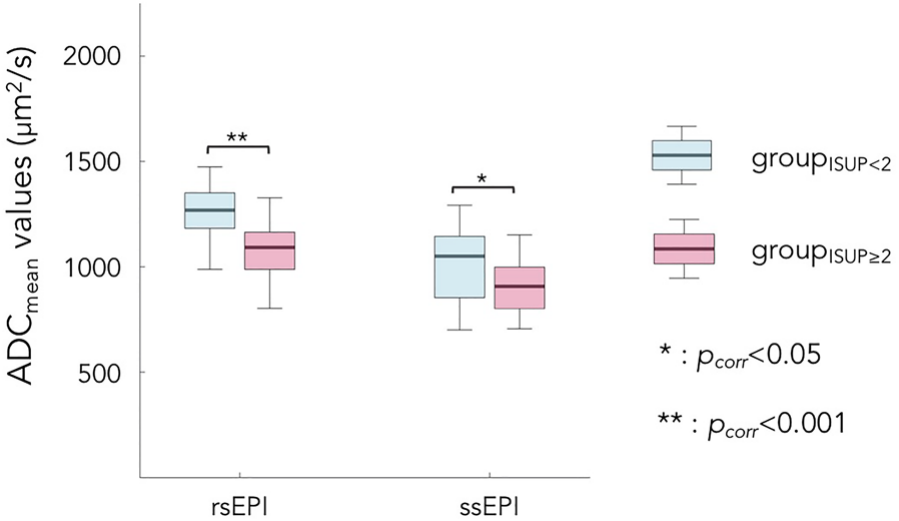
Group-level comparison of ADC_mean_ between the group_ISUP<2_ (light blue) and group_ISUP≥2_ (light red) for the rsEPI DWI sequence (**left**) and the ssEPI DWI sequence (**right**). Bottom and top edges of the boxes indicate the 25 and 75^th^ percentile. Thick middle line indicates the median. Outliers were not represented. Statistical differences are represented by bars along with *p*-value. *Note*: rsEPI = readout-segmented echo-planar imaging, ssEPI = single-shot echo-planar imaging.

Figure 3 shows boxplots of ADC_mean_ values calculated for each ISUP-based group and each DWI sequence.

We observe that for both DWI sequences, the group_ISUP<2_ has significantly higher ADC_mean_ values than the group_ISUP≥2_ (*p* = 2.21⨉10^–8^ for rsEPI, *p* = 0.023 for ssEPI). Table 3 reports the median value and IQR (in μm^2^/s) of each case.

**Table 3:** ADC_mean_ median and IQR values for each ISUP-based group and each DWI sequence.

	group_ISUP<2_ (μm^2^/s, median (IQR))	group_ISUP≥2_ (μm^2^/s, median (IQR))
rsEPI	1269 (1183–1350)	1092 (988–1164)
ssEPI	1050 (854–1144)	907 (801–998)

ISUP, international society of urological pathology; IQR, interquartile range; rsEPI, readout-segmented echo-planar imaging; ssEPI, single-shot echo-planar imaging.

This result was confirmed by an additional analysis with a sub-group of rsEPI patients matched for age and PSA density with the ssEPI group. Indeed, we also observed that group_ISUP<2_ have significantly higher ADC_mean_ values than the group_ISUP≥2_ for the rsEPI DWI sequence (*p* = 8.75⨉10^–5^).

### *Diagnostic accuracy of rsEPI and ssEPI for the ADC*_*mean*_

ROC curves analysis for the diagnostic accuracy of the ADC_mean_ to discriminate group_ISUP<2_ from group_ISUP≥2_ for each DWI sequence show a better discrimination of group_ISUP<2_ from group_ISUP≥2_ using the rsEPI than the ssEPI.

The rsEPI is characterized as a very good classifier with an AUC of 0.84 (0.78–0.9) and an optimal cut-off value of 1232 μm^2^/s associated with a pair of sensitivity–specificity of 97%–63%. The ssEPI showed to be a satisfactory classifier with an AUC of 0.68 (0.54–0.82) and an optimal cut-off value of 1022 μm^2^/s with a pair of sensitivity–specificity of 82%–56%. The two AUCs were significantly different (*p* = 0.039).

When performing ROC curve analysis of matched groups, the rsEPI remains a very good classifier with an AUC of 0.82 (0.71–0.93) with an optimal cut-off value of 1211 μm^2^/s (sensitivity: 95%, specificity: 68%). For the matched group analysis, the two AUCs did not reach significance.

## Discussion

Our study succeeded in showing a better diagnostic accuracy of the rsEPI DWI sequence at discriminating patients with csPCa based on ADC values within the index lesion. In particular, we highlight that the rsEPI DWI sequence (i) enhances significant differences between ISUP-based groups and (ii) has better diagnostic accuracy than the conventional ssEPI for the discrimination of patients with csPCa.

As previously mentioned, the main difference between rsEPI and ssEPI sequences is related to the intrinsic properties of how the *k*-space is filled. ssEPI sequence fills the *k*-space in a single shot, whereas rsEPI sequence partitions the *k*-space into non-overlapping segments in the readout direction. This segmentation has shown to contribute to less magnetic-susceptibility artifacts and T2* blurring^10^, and several studies have already shown a better image quality for PCa imaging using the rsEPI sequence than the conventional ssEPI^11,12^. Our study thus complements these qualitative studies by bringing the additional information, that is, an improved diagnostic accuracy of the rsEPI compared to ssEPI for distinguishing patients with csPCa.

Aside from the intrinsic difference between the rsEPI and the ssEPI described above, another explanation may come from the MRI parameters chosen for each sequence, in particular the lowest *b*-value chosen (*b*-value of 0 s/mm^2^ for the rsEPI, 50 s/mm^2^ for the ssEPI). Indeed, the monoexponential fitting of the rsEPI sequence with the lowest *b*-value of 0 s/mm^2^ takes perfusion effects somehow into account as the intercept is higher than if the lowest *b*-value would have started at a greater *b*-value (such as 50 s/mm^2^). Given the potential microstructures differences as a function of the ISUP grade groups, this may explain why rsEPI (with a lowest *b*-value of 0 s/mm^2^) enables to highlight differences between the two ISUP-based groups. This explanation is in agreement with Riches et al.^19^, who compared the monoexponential and biexponential fitting in different prostatic zones taking into account a large range of *b*-values. They showed that the biexponential model best described the data of tissues located within the PZ and in malignant tumor regions, suggesting the contribution of strong perfusion effects. The fact that perfusion effects are not negligible and should be taken into account in the investigation of PCa reinforces the need to perform biexponential fitting of ADC maps rather than monoexponential fitting as conducted here^20,21^. In addition, it is worth mentioning that the seminal studies disclosing an inverse relationship between ADC values and ISUP-based groups have been obtained using ssEPI sequences with a lowest *b*-value of 0 s/mm^2^^4–6^. It is also important to underscore that the DWI sequence was different according to the corpulence of the patients (rsEPI for non-corpulent patients and ssEPI for corpulent patients). One might argue that the differences observed might thus be attributed to the corpulence of the patients, as corpulent patients lead to less MR signal recorded and thus lower SNR. Although this latter statement is true as we observed a global lower ADC values for the ssEPI compared to the rsEPI, this effect impacts both the group_ISUP<2_ and the group_ISUP≥2_ within the ssEPI sequence, and is therefore present in the two clinical groups. Taken together, our results showed that rsEPI performs better than ssEPI in discriminating patients with csPCa, which could be explained either by the intrinsic design of the rsEPI or by the choice of the lowest *b*-value.

With the rsEPI, we found that the ADC_mean_ is a very good classifier, for which optimal ADC threshold is associated with a high sensitivity and good specificity. This threshold should help radiologists to better select patients with csPCa for prostate biopsy and eventually guide the procedure. Furthermore, current PI-RADS guidelines do not include DWI-derived quantitative parameters for the estimation of PCa probability. We hope that results such as those from the present study will suggest to consider and integrate quantitative DWI parameters including threshold values in PI-RADS guidelines. As a matter of fact, the introduction of such threshold values is reinforced by the number of suspicious lesions described by experienced radiologists that, after biopsy, corresponds to benign lesions (70 out of 105 for rsEPI, 32 out of 54 for ssEPI). Interestingly, when considering matched rsEPI with ssEPI, we observed that the AUC for rsEPI remains a very good classifier but we lost the statistical difference between the two AUCs. This loss of significance is a mere reflection of the smaller sample size of the two datasets (the matched rsEPI and the ssEPI). Smaller dataset means higher variance and thus a lowered *z*-statistic.

Our results can be discussed in the light of two 3T MRI studies: Costa et al.^9^, which investigates the discrimination of ISUP grade 1 from ISUP grade 2 and Boschheidgen et al.^22^, which defines MRI grading for the prediction of PCa aggressiveness. In Costa el al.^9^, the authors computed the ADC from a monoexponential fitting using a ssEPI sequence with a lowest *b*-value of 0 s/mm^2^ and with an additional endorectal coil. Compared to our results, they reported a better AUC (of 0.91) and a lowered optimal threshold (680 μm^2^/s). In Boschheidgen et al.^22^, both ssEPI and rsEPI sequences were used (without additional endorectal coil) with a lowest *b*-value of 0 s/mm^2^. In line with our study, they found lower thresholds for the ssEPI sequence than for rsEPI sequence. A key difference between these two studies and our work is related to the ADC-metric used. While we measured the mean ADC value within the whole lesion volume, these studies have relied on a two-dimensional region-of-interest (2D ROI) of lowest perceived ADC values manually drawn by radiologists. It is therefore expected to have lowered ADC-related thresholds using this latter approach. However, it should also be noted that 2D ROI drawn by radiologists is more prone to possible inter-observer variability^23^ and the use of our ADC-related metric bypasses this possible issue. Other differences with our study could be explained by their larger dataset, the use of the additional endorectal coil (as in^9^), or the different numbers/values of *b*-values, which renders the further comparison with our study difficult.

Our study faces some main limitations. A first main drawback is its retrospective approach which comes along with substantial variability in measurement techniques and suboptimal study population design. A future prospective study with closer MRI parameters between the two DWI (in particular for the *b*-values) and with patients undergoing the two DWI successively (i.e., a paired design) could raise the retrospective issues of our analysis. Secondly, our study has a limited sample size and future works should rely on greater sample size. As a matter of fact, when performing ROC curve analysis, we lost the significant difference between the two AUCs, which is attributable to lowered sample size. Thirdly, our database extends over a period encompassing both PI-RADS version 2.0 and version 2.1. However, these changes (considering DWI within the PZ) relied on precisions about the definition of a PI-RADS score 3 but did not affect the global score assigned to a PZ lesion. We thus expect a marginal impact of this upgrade on our results. Lastly, we only consider index lesions but not non-index lesions. Indeed, ISUP grade groups were solely considered based on the index lesion. Our study disclosed optimal ADC cut-off values to guide csPCa patients towards biopsies only on the basis of the segmentation of the index lesion. We must ensure that no csPCa is found outside the index lesion and that our thresholds do not miss patients for biopsies (i.e., patients having no PCa based on index lesion but do have PCa based on non-index lesion). A future work should test the robustness of our ADC cut-off values against this potential issue. Another interesting future investigation based on our study should investigate to what extent the rsEPI DWI still improved the discrimination of positive from negative lesions in patients with PI-RADS 3 score, as for this category of patients, the medical direction and therapeutic strategy still remains an open debate. Finally, a natural extension of this work would be to translate it on a 1.5T MRI system.

In conclusion, this monocentric retrospective study based on MRI-targeted biopsy has enabled us to highlight that the rsEPI DWI sequence with a lowest *b*-values of 0 s/mm^2^ is best suited for discriminating patients with csPCa within the PZ. Using a rsEPI DWI sequence, a larger number of csPCa may thus be detected to guide MRI-targeted biopsy. The threshold highlighted in our study should now be confirmed using a larger dataset and a multicentric study design to strengthen and reinforce the robustness of our cut-off ADC values.

## Data Availability

The datasets used and analyzed during the current study are available from the corresponding author on reasonable request.
